# Correlation between dietary information sources and knowledge of adequate diets in Eastern China

**DOI:** 10.3389/fpubh.2022.955766

**Published:** 2022-09-28

**Authors:** Bin Cui, Linda Dong-Ling Wang, Fu Rong Wang, Jing Peng, Jian Ying Ma, Xiang Chen, Mei Yin Xu, Jun Ke, Yi Tian

**Affiliations:** ^1^Business School of Yangzhou University, Yangzhou, China; ^2^Jiangsu Key Laboratory of Zoonosis, Yangzhou University, Yangzhou, China; ^3^Jiangsu Co-Innovation Center for Prevention and Control of Important Animal Infectious Diseases and Zoonoses, Yangzhou University, Yangzhou, China; ^4^Institute of Translational Medicine, Medical College, Yangzhou University, Yangzhou, China; ^5^Jiangsu Key Laboratory of Experimental and Translational Non-Coding RNA Research, Yangzhou University, Yangzhou, China; ^6^School of Tourism, Cuisine of Yangzhou University, Yangzhou, China; ^7^School of Public Health of Yangzhou University, Yangzhou, China

**Keywords:** dietary, information sources, knowledge, adequate diets, Eastern China

## Abstract

Knowledge of adequate diets can improve an individual's health status. Although previous studies have identified the main resources from which Chinese people acquire dietary knowledge, it is still unclear whether information sources regarding diets (ISRDs) can increase individuals' knowledge of adequate diets (KAD) and which ISRDs are most effective in conveying KAD to the Chinese population. In this study, we interviewed 4,710 residents in Eastern China regarding their ISRDs and KAD. Descriptive statistics, ANOVA, and multivariate linear regression were used to analyze the effectiveness of different ISRDs in transmitting KAD to Chinese individuals and to determine the relationship between ISRDs and KAD. Results showed that the KAD scores of the respondents were low overall in Eastern China. Providing dietary information through expert lectures, books, newspapers, magazines, and social media could significantly improve the average KAD score of Chinese individuals. Respondents with a greater number of ISRDs were more likely to have higher KAD scores. These findings suggest that the number of ISRDs should be increased. In particular, emphasis should be placed on the role of expert lectures, books, newspapers, magazines, and social media.

## Introduction

The Chinese Nutrition Society defines an adequate diet as a diverse, balanced diet that meets the nutritional and health needs of an individual or group with different health conditions, geographical resources, habits, and beliefs (1). Consuming an adequate diet is associated with a reduced risk of death from all causes (2, 3). A recent research report from the Chinese Nutrition Society showed that consuming an inadequate diet is the leading cause of chronic disease and death in China, which accounted for 3.1 million deaths in 2017 (1).

Dietary knowledge is the basis of individual diet-related behavioral change (4, 5), and better dietary knowledge can improve Chinese individuals' health status (6, 7). Therefore, increasing the population's knowledge of adequate diets (KAD) by various means is an important way to promote a healthy diet and reduce the incidence of chronic disease and death.

Previous studies have found that television, radio, books, newspapers, magazines, and the internet are the main resources from which Chinese people acquire dietary knowledge, and that females, younger individuals, and people with higher levels of education are more likely to actively seek out nutrition and health information (8, 9). However, it is still unclear whether information sources regarding diets (ISRDs) can increase individuals' KAD, which ISRDs are most effective in conveying KAD to the Chinese population, and what sources of dietary information are preferred by different demographic groups.

Eastern China (including Jiangsu, Shandong, Anhui, Zhejiang, and Fujian Provinces and Shanghai Municipality) is a region of the country that is experiencing rapid economic development. The per capita gross regional production of Eastern China was 103,169 Chinese yuan in 2020, which is higher than the national average (10). Therefore, studies performed in Eastern China are useful for guiding decision- and policy-making in the rest of the country.

The objectives of this study were to clarify the effectiveness of different ISRDs in transmitting KAD to Chinese individuals, to determine the relationship between ISRDs and KAD, and to clarify the sources of dietary information that are preferred by different demographic groups.

## Methods

### Sampling

The current study was conducted in Eastern China (including Jiangsu, Shandong, Anhui, Zhejiang, and Fujian Provinces and Shanghai Municipality). Two cities were randomly selected from each province. In each selected city, two residential areas were randomly selected (four residential areas were randomly selected from Shanghai Municipality) for sampling. Two hundred residents were recruited from each residential area. Five trained research assistants conducted face-to-face interviews with the residents based on a standardized questionnaire by asking questions and taking notes. Interviews were conducted at the entrance of each residential area in the afternoon at the end of the normal work day. The average interview lasted about 10 min for each participant. Upon completion of the interview, each participant received a small gift worth about 5 Chinese yuan.

A total of 4,710 participants completed interviews, for a response rate of 98.1%. Only 90 residents refused to participate, citing lack of time or other, unstated reasons.

### Ethics, consent, and permissions

The survey was conducted from March to June 2021 following ethical approval from Yangzhou University. Potential survey participants were first provided with an explanation of the study and asked for their consent to participate. Those who agreed to participate completed a face-to-face interview using a standardized questionnaire (see Appendix). The questionnaire was fully anonymous and did not collect any personal, identifying information.

### Study instrument

The questionnaire was divided into two sections. The first section was designed to collect socio-economic information, including gender, age, level of education, monthly family income, and province or municipality, as well as the ISRDs that residents were exposed to. To obtain a complete picture of the ISRDs that individuals could be exposed to, we conducted a pilot survey that involved interviewing 15 residents of different ages and genders. Based on this survey, we generated a list of possible ISRDs (e.g., expert lectures, books, newspapers, magazines, television, radio, friends or relatives, the internet, social media, TikTok, and health care product sales staff). Interviewing five other residents did not yield any additional ISRDs, so we considered the ISRD list to be complete. The list was multiple-choice, and respondents were asked to indicate all their actual ISRDs.

The second part of the survey addressed KAD. To minimize respondents' time burden, and thereby improve the completion rate of the questionnaire, we selected seven main dietary knowledge items from the “The Chinese Dietary Guidelines” (2016) enacted by the Chinese Nutrition Society. To ensure that each question was easily understood, clear, and answerable, we listed daily dietary intake quantities in grams or milliliters according to “The Chinese Dietary Guidelines” (2016) and provided four possible answers to each question, only one of which was correct (see Appendix).

### Data analysis

First, descriptive statistics were used to calculate the percentage of correct answers to each item in the KAD part of the questionnaire, as well as the percentages of selected and unselected ISRDs.

Second, each respondent's score (out of 100) for the KAD items was calculated, and then ANOVA was used to compare average KAD scores with each ISRD (*P-values* of < 0.05 were considered significant).

Third, multivariate linear regression was employed to examine the socio-economic variables associated with the number of ISRDs, as well as both the socio-economic variables and the number of ISRDs associated with KAD scores.

SPSS 20.0 software was used to perform all of the statistical analyses described above.

## Results

### Study population demographics

The demographic characteristics of the study participants are presented in Table 1. Of the 4,710 respondents, 56.5% were female, and the majority of respondents were aged between 36 and 55 years (47.5%). In total, 47.3% of the study participants had an educational level of undergraduate college or above. A monthly family income of between 5,000 and 9,999 Chinese yuan was reported by the highest proportion of respondents (32.9%). The sample was representative of the demographic structure of the general population of Eastern China, as reported in the China Statistical year book (2020) (10).

**Table 1:** Respondent characteristics (*N* = 4,710).

**Characteristics**	***N***	**%**
**Gender**
Male	2,049	43.5
Female	2,661	56.5
**Age**
≤ 20 years	885	18.8
21–35	1,293	27.5
36–45	1,327	28.2
46–55	908	19.3
56–65	254	5.4
66–75	34	0.7
≥76 years	8	0.2
**Education**
Primary or below	92	2.0
Junior high school	655	13.9
Senior high school	905	19.2
Three-year college	830	17.6
Undergraduate college	1,789	38.0
Postgraduate and above	439	9.3
**Monthly family income (Chinese Yuan)**
≤ 5,000	1,301	27.6
5,000–9,999	1,548	32.9
10,000–19,999	1,099	23.3
20,000–39,999	464	9.9
40,000–80,000	158	3.4
≥80,001	140	3.0
**Province (or Municipality)**
Jiangsu	913	19.4
Shandong	801	17.0
Anhui	953	20.2
Shanghai municipality	618	13.1
Zhejiang	709	15.1
Fujian	716	15.2

### Answers regarding knowledge of adequate diets

To assess KAD levels, the study participants were asked seven questions regarding appropriate daily intake of a variety of staple foods. Regarding the seven KAD items (Table 2), daily salt intake had the highest percentage of correct answers (41.2%), followed by daily intake of vegetables (34.7%). However, the type of food eaten every day had the lowest percentage of correct answers (15.9%). Taken together, the responses indicated that, overall, KAD is low in this population.

**Table 2:** Number of correct answers to each knowledge of adequate diets item (*N* = 4,710).

**Dietary knowledge item**	***N***	**%**
Daily use of cooking oil 25–30 g	1,539	32.7
Daily salt intake not to exceed 6 g	1,939	41.2
Daily sugar take amount not to exceed 50 g	1,243	26.4
Daily intake of milk and dairy products 300 g	1,015	21.5
Daily water intake 1,500–1,700 ml	1,276	27.1
Daily intake of vegetables 300–500 g	1,635	34.7
Eat at least 12 types of food a day	751	15.9

### Differences in dietary knowledge scores among different information sources regarding diets

Regarding ISRDs (Table 3), 54.9% of respondents reported that they get information about reasonable diets from books, newspapers, and magazines; 51.9% reported obtaining dietary information from social media; 49.8% reported obtaining information from friends or relatives; and only 6.6% reported revising dietary information from health care product sales staff.

**Table 3:** Origin of information regarding diet and ANOVA analysis of knowledge of adequate diets score means among different information sources regarding diets (*N* = 4,710).

**Information sources regarding diets**	**Group**	***N* (%)**	**Knowledge score mean**	***F***	***P-*value**
Expert lectures	No	3,453 (73.3)	25.94	158.55	0.000
	Yes	1,257 (26.7)	36.86		
Books, newspapers, and magazines	No	2,126 (45.1)	24.24	118.30	0.000
	Yes	2,584 (54.9)	32.66		
Television and radio	No	2,859 (60.7)	28.27	3.54	0.060
	Yes	1,851 (39.3)	29.77		
Friends or relatives	No	2,363 (50.2)	29.38	1.79	0.181
	Yes	2,347 (49.8)	28.33		
Social media	No	2,266 (48.1)	27.61	9.49	0.002
	Yes	2,444 (51.9)	30.01		
Health care product sales staff	No	4,399 (93.4)	28.97	1.16	0.281
	Yes	311 (6.6)	27.28		

Results from the ANOVA analysis showed that there were significant differences in the mean KAD score depending on whether the respondent received dietary information from expert lectures (*F* = 158.55, *P* < 0.01), books, newspapers, and magazines (*F* = 118.30, *P* < 0.01), and social media (*F* = 9.49, *P* < 0.01) compared with other sources (Table 3).

### Determinants of different information sources regarding diets, number of information sources regarding diets, and knowledge of adequate diets score

The results from the logistic regression analysis (Table 4) showed that female respondents and older respondents were less likely to obtain dietary information from Expert lectures, whereas highly educated and high-income respondents were more likely to obtain dietary information from Expert lectures and Books, newspapers, and magazines. High-income respondents were less likely to obtain dietary information from Television and radio. Female, older, and high-income respondents were more likely to obtain dietary information from Friends or relatives, whereas highly educated respondents were less likely to obtain dietary information from Friends or relatives. Female and high-income respondents were more likely to obtain dietary information from Social media, whereas highly educated respondents were less likely to obtain dietary information from Social media. High-income respondents were more likely to obtain dietary information from Health care product sales staff, whereas older respondents and highly educated respondents were less likely to obtain dietary information from Health care product sales staff. On the other hand, the results from the linear regression analysis (Table 4) showed that gender, level of education, and income were significantly and positively associated with number of ISRDs, while age was significantly and negatively associated with number of ISRDs. Age, level of education, income, and number of ISRDs were significantly and positively associated with KAD score.

**Table 4:** Multiple regression coefficients (odds ratio, standardized β) for socio-economic variables in terms of different information sources regarding, reasonable diets (ISRDs) associated with knowledge of adequate diets (KAD) score.

**Predictor**	**Logistic regression analysis regarding different**						**Linear regression**	
**variables**	**information sources regarding diets**						**analysis**	
	**Expert lectures**	**Books, newspapers, and magazines**	**Television and radio**	**Friends or relatives**	**Social media**	**Health care product sales staff**	**Number of ISRDs**	**KAD score**
	**OR**	**OR**	**OR**	**OR**	**OR**	**OR**	**β**	**β**
Gender (female)	0.822**	1.079	1.082	1.424**	1.328**	1.234	0.058**	−0.004
Age	0.810**	1.014	1.047	1.096**	0.791**	0.697**	−0.066**	0.098**
Level of education	1.174**	1.236**	0.986	0.934**	0.977	0.837**	0.039**	0.125**
Income	1.168**	1.090**	0.947*	1.063*	1.120**	1.130**	0.076**	0.049**
Number of	-	-	-	-	-	-	-	0.119**
information
sources

^*^P < 0.05, ^**^P < 0.01.

“-” indicates no data.

OR, odds ratio; β, standardized coefficient.

## Discussion

The present study offers insights into ISRDs and KAD scores among residents of Eastern China, as well as into the relationship between ISRDs and KAD. In this study, we found that Expert lectures, Books, newspapers, magazines, and Social media were the most effective ISRDs for conveying KAD to residents of Eastern China, that different demographic groups prefer different information sources, and that a greater number of ISRDs correlated with higher KAD scores.

The KAD scores of the respondents in Eastern China were low overall. This is consistent with data from the China Health and Nutrition Survey (CHNS) conducted in 2015 in nine Chinese provinces and three municipalities, which reported that only 34.3% of the participants had adequate dietary literacy (6). Our findings indicate that the KAD level of residents in Eastern China has not improved significantly in recent years. This suggests that increasing the KAD of residents remains an important target for dietary intervention and education.

The survey results showed that Books, newspapers, and magazines, Social media, and Friends or relatives were the main information sources from which respondents acquired dietary knowledge. This is essentially consistent with the 2015 CHNS report (8), as well as with another survey conducted in Beijing in 2015 (9). Thus, the findings from the current study suggest that providing dietary information through Expert lectures, Books, newspapers, and magazines, and Social media significantly improves the average KAD score of Chinese individuals. In terms of the most effective ISRDs for conveying KAD to residents of Eastern China, the survey found that young respondents and respondents with a high income level were more likely to obtain dietary information from Social media, and that highly educated respondents were more likely to obtain dietary information from Books, newspapers, and magazines. This is consistent with a survey conducted in Beijing in 2015 (9). However, our survey further revealed that female respondents were more likely to obtain dietary information from Social media, and that respondents with a high income level were more likely to obtain dietary information from Books, newspapers, and magazines. Male respondents, young respondents, and highly educated and higher income respondents were more likely to obtain dietary information from Expert lectures. Therefore, when disseminating dietary knowledge through the above information sources, more attention should be paid to elderly, low-income, and less educated individuals.

The survey results showed that female respondents, young respondents, and respondents with a high level of education were more likely to be exposed to a greater number of ISRDs. This is consistent with a previous study conducted in Beijing in 2015, which found that females, young people, and individuals with a higher level of education are more likely to actively seek out nutrition and health information (9). People who actively seek out nutrition and health information inevitably have more access to ISRDs; however, more convenient ISRDs are fundamental to improving residents' knowledge. In the current study, we found that individuals with higher income were exposed to a greater number of ISRDs, possibly because high-income groups are more likely to have access to information on healthy eating (11). Therefore, to improve residents' KAD levels, it is necessary to provide more ISRDs while increasing residents' attention to dietary nutrition and health.

Older respondents and respondents with high levels of education and higher income were more likely to have a higher KAD score. This is consistent with previous studies conducted in Chinese urban adults (12), Swiss individuals (13), Belgians (14), and Iranians (15) that found that an individual's level of nutrition knowledge mainly depends on his/her education, age, and income. While nutrition knowledge focuses more on the nutrients provided by food and their association with disease, and dietary knowledge is more concerned with the importance of an adequate diet, both dietary knowledge and nutrition knowledge emphasize the importance of healthy eating (16). In the current study, we found that respondents with a greater number of ISRDs were more likely to have a higher KAD score. This further proves that a greater number of ISRDs plays an important role in improving residents' knowledge scores.

This study also found that young respondents have more ISRDs, while older respondents have higher KAD scores. This could be because younger people have a lower perception of being at risk of chronic disease than older people, because chronic diseases primarily affect older individuals (17), thus prompting older people to pay more attention to KAD than younger people. However, further research is needed to clarify the actual reasons for this observation.

This study had several limitations. First, the cross-sectional design excluded causal inference. However, the correlation among the different variables discussed in this study suggests that the data offer a plausible explanation for cause and effect in this population. Second, this study was limited to Eastern China and therefore cannot be considered representative of residents throughout China. In the future, surveys should be conducted in an expanded study area. However, the contribution of this study to the literature is to confirm the relationship between ISRDs and KAD and to find that expert lectures, books, newspapers, and magazines and social media were most effective in conveying KAD to the Chinese population.

## Conclusions

In this study, we found that residents of Eastern China have low KAD scores. According to our study, ISRDs and KAD are closely related; therefore, to improve KAD scores, the number of ISRDs should be increased. In particular, emphasis should be placed on the role of Expert lectures, Books, newspapers, and magazines, and Social media to improve KAD, and interventions should target the elderly, low-income individuals, and individuals with a low level of education.

## Data availability statement

The original contributions presented in the study are included in the article/Supplementary material, further inquiries can be directed to the corresponding author.

## Author contributions

BC: conceptualization, methodology, investigation, data curation, and writing—original draft. LW: software, investigation, and writing—review and editing. FW, JP, JM, XC, MX, JK, and YT: investigation and writing—review and editing. All authors have read and agreed to the published version of the manuscript.

## Funding

This research was supported by the Open Project Program of Jiangsu Key Laboratory of Zoonosis (No: R2004).

## Conflict of interest

The authors declare that the research was conducted in the absence of any commercial or financial relationships that could be construed as a potential conflict of interest.

## Publisher's note

All claims expressed in this article are solely those of the authors and do not necessarily represent those of their affiliated organizations, or those of the publisher, the editors and the reviewers. Any product that may be evaluated in this article, or claim that may be made by its manufacturer, is not guaranteed or endorsed by the publisher.
