# Supplementary material for: Correlation between dietary information sources and knowledge of adequate diets in Eastern China
Source: Front Public Health. 2022 Sep 28;10:955766. doi: 10.3389/fpubh.2022.955766 (PMC9554651; doi:10.3389/fpubh.2022.955766)
Supplement: Supplementary file 1 [file Data_Sheet_1.docx]

**Appendix**

Questionnaire

**Gender**

□ Male

□ Female

**Age**

□ ≤20 years

□ 21–35

□ 36–45

□ 46–55

□ 56–65

□ 66–75

□ ≥76 years

**Education**

□ Primary or below

□ Junior high school

□ Senior high school

□ Three-year college

□ Undergraduate college

□ Postgraduate and above

**Monthly family income (Chinese Yuan)**

□ ≤5000

□ 5000–9999

□ 10000–19999

□ 20000–39999

□ 40000–80000

□ ≥80001

**Province (or Municipality)**

□ Jiangsu

□ Shandong

□ Anhui

□ Shanghai Municipality

□ Zhejiang

□ Fujian

**Origin of information regarding reasonable diets**

□ Expert lectures

□ Books, newspapers, and magazines

□ Television and radio

□ Friends or relatives

□ Social media (e.g., TikTok)

□ Health care product sales staff

**How many grams of cooking oil does the Chinese Nutrition Society suggest that each adult consume on a daily basis?**

□ 5–10 grams

□ 25–30 grams

□ 40–50 grams

□ Don’t know

**What is the maximum daily salt intake recommended by the Chinese Nutrition Society for adults?**

□ 3 grams

□ 6 grams

□ 10 grams

□ Don’t know

**What is the maximum daily sugar intake recommended by the Chinese Nutrition Society for adults?**

□ 40 grams

□ 50 grams

□ 60 grams

□ Don’t know

**How much milk and dairy products does the Chinese Nutrition Society suggest that adults consume on a daily basis?**

□ 100 grams

□ 200 grams

□ 300 grams

□ Don’t know

**How much water does the Chinese Nutrition Society suggest that each adult drink per day?**

□ 1000–1200 ml

□ 1500–1700 ml

□ More than 2000 ml

□ Don’t know

**What is the minimum daily intake of vegetables suggested by the Chinese Nutrition Society for adults?**

□ 100–200 grams

□ 200–300 grams

□ 300–500 grams

□ Don’t know

**At least how many types of food are recommended for consumption by adults by the Chinese Nutrition Society every day?**

□ 10 types

□ 12 types

□ 15 types

□ Don’t know
